# Cases of Interspecies Transmission of Influenza A Virus from Swine to Humans

**DOI:** 10.3390/vetsci12090873

**Published:** 2025-09-09

**Authors:** Nailya Klivleyeva, Tatyana Glebova, Nurbol Saktaganov, Richard Webby

**Affiliations:** 1 The Research and Production Center for Microbiology and Virology, Almaty 050010, Kazakhstan; taty1962@mail.ru (T.G.); nsaktaganov1984@mail.ru (N.S.); 2 Department of Infectious Disease, St. Jude Children’s Research Hospital, Memphis, TN 38105-3678, USA; richard.webby@stjude.org

**Keywords:** influenza viruses, swine influenza, zoonotic infection, transmission, epizootology, reassortment

## Abstract

Influenza A viruses infect many animal species and have great zoonotic potential. The epidemiology of swine influenza worldwide is of particular importance because the pig can act as a “mixing vessel” where avian and human influenza viruses can undergo genetic reassortment, creating new viruses. Zoonotic transmission allows new strains of influenza A viruses to be introduced into the human population, potentially causing the next influenza pandemic. The dynamic nature of swine influenza viruses poses challenges to both the swine industry and public health as a source of zoonotic infection. Human infections with swine influenza viruses are regularly reported. Many of these zoonotic events have occurred through close contact between humans and pigs, particularly at agricultural fairs, which have become a source of emerging of swine-origin influenza A viruses. These unique pig–human interfaces have caused the majority of human infections with variant influenza A viruses. In this review, we examine zoonotic spread of influenza A viruses of swine origin, transmission of influenza viruses from pigs to humans worldwide, and the reasons for the emergence of zoonoses.

## Simple Summary

The particular susceptibility of pigs to infection with avian and mammalian influenza viruses allows them to be considered as a mixing vessel where reassortment occurs as a result of coinfection, which promotes the expansion of viral diversity. Although influenza viruses have low pathogenicity in pigs, the outcome of coinfection with other pathogens can be severe, even fatal. Globally, pigs play a key role in the transmission of influenza A viruses between species and the emergence of zoonotic influenza and pandemic viruses. Although human infection with zoonotic influenza viruses is less common than infection with seasonal influenza viruses, there is global concern that these zoonotic viruses may acquire mutations in animals or humans and facilitate efficient animal-to-human trans-mission or persistent human-to-human transmission. The possibility of genetic exchange and interspecies transmission of influenza A viruses is facilitated by the proximity of pig and poultry farms, the production and trade of farm animals and animal products, as well as people working on pig farms. Therefore, ongoing surveillance of both pigs and humans, especially those at increased risk of complications from infection due to exposure at the animal–human interface, and limiting human–pig contact can help prevent severe infections and reduce the public health burden.

## 1. Introduction

Currently, more than 1400 species of infectious organisms are known to be pathogenic to humans; of these, more than 60% are zoonotic infections transmitted from animals to humans [1,2]. The emergence of new pathogens poses a constant threat to human health.

In the last decade, numerous human infections with zoonotic diseases have been reported, including Ebola virus, Nipah virus, Hendra virus, severe acute respiratory syndrome (SARS) coronavirus, Middle East respiratory syndrome (MERS) coronavirus, and COVID-19. Although these zoonotic viruses cause illness and death in humans, many of them lack the ability to transmit sustainably from person to person and therefore do not pose a global health threat. However, respiratory viruses have the potential to adapt to replicate and spread between humans and cause pandemics, and the drivers and risk factors for the transmission of influenza A viruses (IAVs) across species barriers are poorly understood.

Influenza viruses (IVs) belong to the Orthomyxoviridae family and are negative-sense RNA viruses with a segmented genome. IVs are classified into four types (A, B, C, and D) based on genetic and antigenic differences. Of all types of IVs, IAVs are the most important pathogens that pose a significant risk of zoonotic infection, host shifts, and the emergence of pandemic viral variants.

IAVs are capable of infecting a wide range of animals, including humans, wild and domestic birds, pigs, horses, seals, whales, dogs, mink and other mammals [3]. However, the natural reservoirs of IAVs are wild birds, especially Anseriformes (ducks, geese and swans) and Charadriiformes (gulls, terns and surfbirds) [4]. IAVs are unique in their ability to evolve and adapt after crossing the species barrier to replicate and spread to other individuals within the new species. Novel human-adapted IAVs have emerged several times over the past 100 years, causing pandemics [5]. Each pandemic virus emerged in a different way, making it difficult to understand how zoonotic IAVs adapt to humans. IAVs are also unique in the frequency with which they transmit across species. As knowledge accumulates, it is recognized that the host–virus interaction is critical for the transmission of IAVs to a new host. IAVs infections result in a wide range of clinical outcomes that depend on both the virus strain and viral load, host species, host immunity, and environmental factors. The lack of consistent host-switching events for IAVs from natural reservoirs (pigs, poultry) to humans and domestic animals further highlights the complexity of the process and severely limits the ability to predict the future interspecies transmission events associated with pandemic risk.

The ability of swine influenza viruses (SIVs) to spread bidirectionally from animals to humans (zoonosis) and from humans to animals (reverse zoonosis) leads to coinfection, which contributes to the expansion of viral diversity. Many animal species are susceptible to reverse zoonotic transmission of IVs, particularly those in close contact with humans (minks, ferrets, seals, dogs, cats) and certain birds (turkeys, chickens, quails, and ducks). These animals, due to the presence of both α-2,6-SA (the receptor preferred by human influenza viruses) and α-2,3-SA (the receptor preferred by avian IVs) receptors in their tissues, may also be considered potential vessels for mixing during coinfection with human and avian IVs. However, pigs are considered to be particularly susceptible to strains from both avian and human sources. Historically, coinfection of pigs with human, swine, and avian IVs has resulted in multiple viral reassortment events. A striking example is the 2009 pandemic H1N1 virus (pdm09), which arose as a result of such reassortments [6]. Most SIVs are reassortants combining genes from swine, avian, and human viruses. This supports the basic idea that pigs can serve as a “mixing vessel” for human and avian IVs [7,8].

Since complex virus–host interactions are influenced by host shift/adaptation, transmissibility and pathogenicity/virulence [5,9], ongoing surveillance of infectious diseases and prediction of which of the various zoonotic IAVs may cross the species barrier and enter the human body remains a pressing issue worldwide.

## 2. Materials and Methods

The literature search was conducted using the Scopus, Web of Science, Google Scholar, PubMed and CyberLeninka/eLIBRARY.ru databases with no publication date restrictions. The following keywords and phrases were used as literature search criteria: “influenza viruses”, “evolution and interspecies transmission of influenza A virus”, “swine flu”; “reassortment”; “epizootology”; “zoonotic infection”; “transmission”, “swine flu in humans”, “swine flu in humans”, “evolution and interspecies transmission of influenza A virus”, “interspecies transmission of influenza A virus from pigs to humans”, “transmission of swine influenza virus to humans”. The references obtained were checked for compliance with the topic of the article and references containing the full text of the articles or informative abstracts were selected for further work. The articles were limited to English and Russian. Considering that English is the dominant language in scientific literature and is essentially projected as an international language that facilitates the exchange of knowledge between scientists around the world, it was more convenient to search in English.

**Figure 1:**
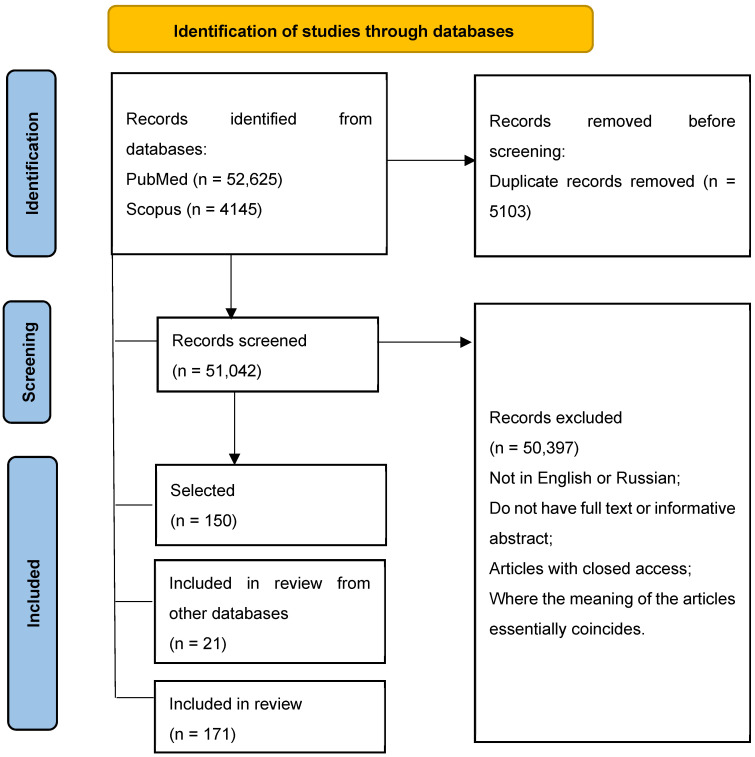
PRISMA flow diagram of the literature search process.

To conduct a comprehensive analysis of the relevant literature, no additional restrictions such as sample size or journal quality were introduced. All the above-mentioned criteria for inclusion and exclusion of articles were defined apriori. All relevant studies were reviewed, including books, government publications and reports, and doctoral dissertations. A flow diagram was constructed to better demonstrate the selection and exclusion criteria for bibliographic sources (Figure 1).

A total of 52,625 studies were identified for screening, of which 5103 were duplicates. 50,397 articles from the search were found to be irrelevant (not meeting the pre-established inclusion criteria) based on title and abstract, language restrictions, lack of full text or informative abstract; and articles with closed access.

The results based on the analysis of 171 publications on the cases of interspecies transmission of influenza A virus from swine to humans are presented below.

## 3. Results

### 3.1. Influenza A Viruses, Their Evolution and Interspecies Transmission

**Figure 2:**
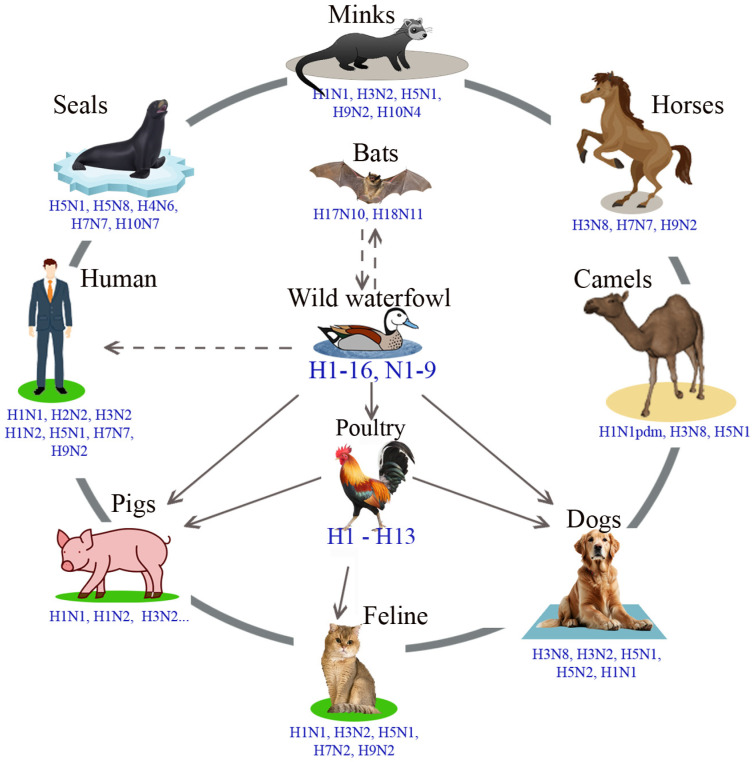
Interspecies transmission of IAVs (H1–H18 and N1–N11). H—hemagglutinin subtype; N—neuraminidases subtype.

The IAVs genome consists of eight RNA segments—PB2, PB1, PA, hemagglutinin (HA), NP, neuraminidase (NA), M, and NS [10]. Surface antigens (HA and NA) are the most variable structural components of the virion and play a key role in the initial stages of cellular infection [11]. Based on the antigenic properties of HA and NA, IAVs are classified into subtypes. To date, 18 HA subtypes (H1–H18) and 11 NA subtypes (N1–N11) have been identified. Most IAVs subtypes have been identified in numerous possible combinations in waterfowl populations [4,12], with the exception of H17, H18, N10, and N11, which have only been found in bats [13,14,15]. Wild waterfowl are considered the natural reservoir of IAVs. Since significant interspecies transmission of IVs occurs in waterfowl, they play a central role in the ecology of IAVs (Figure 2). In contrast, influenza viruses H17, H18, N10, and N11, isolated from bats, have not been detected in any other animal order, and it is unknown whether these viruses are able to cross species barriers. Only a limited number of subtypes (HA H1 and H3 in combination with NA subtypes N1 and N2) have been identified in mammals and have caused epidemic and pandemic influenza in humans and circulate in both humans and pigs [16].

The spread of avian IAV infection has been periodically observed in populations of terrestrial birds, such as domestic turkeys or chickens [17,18]. Sometimes, avian IAVs are able to cross the interspecies barrier and cause epizootic and panzootic diseases in various mammalian species [19,20,21]. Most often, the epidemic process involves people [22,23,24,25,26,27], pigs [24,28,29,30,31,32,33], horses [34,35,36], seals [24,37,38], and minks [39,40]. In recent years, influenza infection has been reported in dogs and cats [41,42]. Thus, one of the most important ecological and epidemiological characteristics of IAV is interspecies transmission [43,44].

For efficient replication and transmission of IAVs, the interaction between HA and NA is critical. The coordinated interaction of HA and NA causes IAVs to bind to host cell receptors. HA is responsible for binding to sialic acid receptors on the host cell surface, initiating infection. At the same time, NA cleaves sialic acid, facilitating the release of newly formed viral particles from the cell and allowing them to infect other cells.

**Figure 3:**
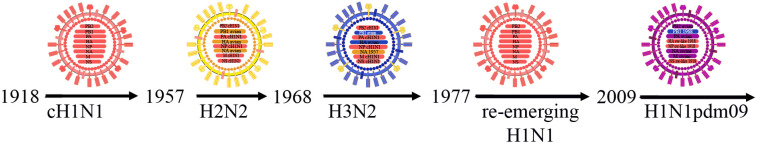
Pandemic cycles of human influenza A virus.

IAVs evolve very rapidly due to antigenic drift and antigenic shift. Antigenic drift, which is responsible for human seasonal influenza viruses, occurs through the gradual accumulation of mutations in the HA and NA surface proteins, leading to antigenic changes in IAVs. Antigenic shift, or reassortment, occurs due to the segmented nature of the IAVs genome. When a host is simultaneously infected with two genetically different IAVs, an exchange of genetic material occurs, what can contribute to the emergence of different recombinant viruses. The accumulation of mutations during replication leads to amino acid replacement that can affect preexisting immunity, host range, virulence, and other factors [45]. Sustained human-to-human transmission of a virus to which a significant part of the population lacks immunity can lead to a pandemic, where large numbers of human cases occur simultaneously on a community-wide scale [20,46,47]. Examples of antigenic shift include the occurrence of five influenza pandemics in human history: the Spanish flu A(H1N1) of 1918, the Asian flu A(H2N2) of 1957, the Hong Kong flu A(H3N2) of 1968, the Russian flu A(H1N1) of 1977, and the pandemic A(H1N1) of 2009 [28,48] (Figure 3).

The large-scale and uncontrolled spread of IAVs is primarily due to their variability, based on point mutations characteristic of RNA-containing viruses, and on gene reassortment [49]. Both mutation and reassortment are associated with interspecies transmission and emergence of IAVs. Thus, it has been shown that mutation led to the transmission of equine IAVs to dogs [50], and recombination led to the emergence of the H1N1 virus in pigs, which caused the 2009 influenza pandemic [51].

In case of combined infection, due to reassortment viral progeny contain different combinations of viral segments from different parent viruses. Thus, reassortment has evolutionary significance because it can create new genomic constellations. The rate of reassortment is influenced by the degree of viral diversity [52]. Since the greatest number of subtypes are found in wild birds, particularly in Anseriformes, recombination is most often found in them [53,54]. In addition, reassortment is of great importance for the emergence of highly pathogenic IAVs of avian influenza in poultry. Thus, it has been shown that IAVs H5Nx and H7N9 appeared as a result of reassortment, since the internal genes of IAVs H9N2, when interacting with various HA/NA subtypes, can form both H5N1 and modern H7N9 viruses [55].

The ecology of IAVs depends on the adaptation of the virus to its host. The main factor determining the host range is the receptor molecule on the surface of the host cell. Different influenza A virus subtypes have evolved to preferentially bind different types of sialic acids. Because of their ability to efficiently bind to and replicate in mammalian cells, influenza A virus subtypes such as H1 and H3 have become dominant in mammals. IAVs bind to sialic acid (N-acetylneuraminic acid), which is linked by an α-glycosidic bond to the terminal galactose residues of the carbohydrate chains of glycoproteins and glycolipids [56,57,58]. Thus, it has been established that avian influenza viruses bind to α-2,3-linked sialic acid, seasonal strains of human IAVs recognize α-2,6-linked sialic acid, and pig tissues express both α-2,6-SA (the receptor preferred by human IAVs) and α-2,3-SA (the receptor preferred by avian IAVs) [7]. To infect and replicate in mammals, including pigs, the H1 and H3 subtypes have evolved to bind efficiently to their alpha-2,6-linked sialic acids. In addition, their ability to evolve and evade the host immune response, especially through antigenic drift, allows them to persist and spread in mammalian populations. Genetic reassortment between human and/or avian and/or swine IAVs may occur in the body of pigs.

There are a number of hosts that carry both types of receptors (avian and human) and can act as potential mixing vessel hosts (wild and domestic birds, humans and various mammalian species); however, pigs are the historically established vessel host for the formation of zoonotic IVs due to receptor expression [8]. Swine become periodically infected with seasonal human IVs through reverse zoonosis, in which H1 and H3 viruses become established, leading to further evolution and diversification and making pigs host to a diverse population of enzootic human IVs [59].

The evolution of swine IAVs is complex and is the result of multiple interspecies transmissions and reassortments that occurred independently on different continents [44,60]. In North America, the classical swine IAV (cIAV) lineage, introduced into pigs from humans during the 1918 pandemic, has circulated for over 70 years. There is evidence of co-circulation of cIAV and human H3N2 in swine, which led to the emergence of a double reassortant (swine–human) and a triple reassortant (swine–human–bird) [61,62]. Further reassortment led to the emergence multiple lineages, each of which carries internal genes of triple reassortant [61,62]. Due to the high degree of reassortment of IAVs in pig populations, some of these viruses may have pandemic potential [57,63,64,65,66]. However, there is no specific evidence for the role of pigs in the human pandemics of 1918, 1957, and 1968, with the exception of the 2009 H1N1 IAV pandemic [67].

While in North America and Europe, a high risk of IAV transmission and spread is associated with pig movements, including both domestic transport and international export [65], in Asia, a high risk of reassortment between avian and pigs IAVs is observed. This is due to the close proximity of pigs, poultry and wild birds, as well as poor biosecurity measures in the pig production system [68]. Influenza infections in pigs are generally mild, causing low mortality. This gives the misconception that influenza does not pose a significant economic risk to the pig industry. As a result, influenza infection is often underestimated, and the virus becomes enzootic and continually evolves, especially in high-density production environments [69,70].

Therefore, there is an urgent need for ongoing monitoring studies to understand the complex dynamics of the evolution and spread of IAVs worldwide.

### 3.2. Transmission of the Swine Influenza Virus to Humans

Of greatest importance to humans are IAVs, which are freely distributed and cause significant morbidity and mortality. Unlike birds and most mammals, humans are susceptible to infection by three types of influenza viruses: A, B, and C. There is also serological evidence of Influenza D virus circulation (which was first isolated from a pig with respiratory symptoms in 2011 in the US) in human populations, particularly in veterinarians working with swine [71,72,73,74,75]. Type C viruses are less common in the human population and do not cause severe disease compared with types A and B. Infections with type A and type B viruses result in mild to severe disease in human populations. IAVs have been responsible for at least five pandemics reported since the early 20th century.

**Figure 4:**
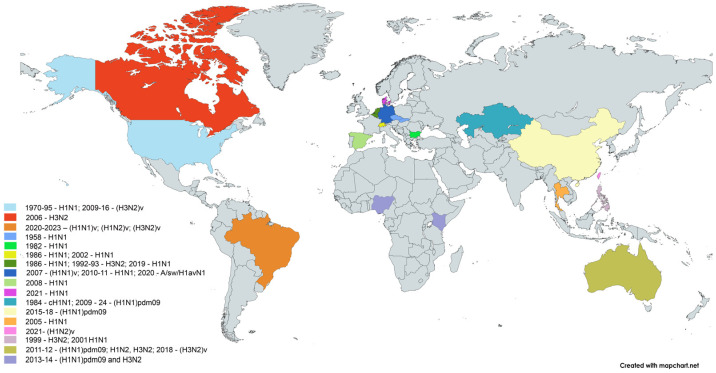
Map of sporadic cases of interspecies transmission of influenza A viruses from pigs to humans. The world map was created online at https://mapchart.net (accessed on 21 August 2025).

Human infection with SIVs occurs through close contact between pigs and humans, particularly on pig farms or in slaughterhouses. Although there are numerous reports of pig-to-human transmission of IAVs, the incidence of human infection with SIVs is much lower than with avian influenza viruses. Figure 4 shows a map of sporadic cases of interspecies transmission of IAVs from pigs to humans.

The first case of human infection with influenza from a pig was identified three years before the isolation of SIVs in 1931 [76]. Robert Shope in the 1930s transmitted the infectious agent from diseased pigs to healthy animals and demonstrated that the human and swine pathogens were closely related because human serum neutralized the swine virus [76,77]. This classic SIV cH1N1 continues to circulate in pigs in Asia and the Americas.

The continents and countries where cases of influenza virus transmission from pigs to humans have been recorded are presented in Table 1.

#### 3.2.1. Transmission of the Swine Influenza Virus to Humans in America

Based on the results of serological and virological studies, by 1970 there was convincing evidence that people were infected with the SIVs by contact with pigs. Thus, an analysis of blood serum conducted in 1970 in the United States showed that antibodies to the SIVs strains isolated by that time were found in 45% of people working in slaughterhouses and quite often in veterinarians [81]. In 1974, the SIV was isolated from the lung of a boy who had contact with pigs five days before his death [82,83]. Antibodies to the virus were also found in pigs. This confirmed the assumption that people could be infected with SIVs.

Following the isolation of SIV in 1974, sporadic cases of SIV disease continued to be reported in the United States. Until 1998, all human cases of SIVs in the United States were caused by classical SIV (cH1N1). In 1975, four cases of cH1N1 SIVs were serologically detected in Wisconsin, Virginia, and Tennessee [83,84,85,86]. Serologic results indicated that isolated cases were infected with an SIV similar to A/New Jersey/1976, which was the cause of a subsequent outbreak of SIVs in humans in 1976. This was the first large-scale human-to-human transmission of SIV when a swine variant of IAV with HA H1 (A/New Jersey/18/76) circulated among military recruits at Fort Dix, New Jersey, in 1976 [87,88,89]. In a review article by Myers et al. Numerous cases of human infection with SIVs have been documented since that time [78,90].

Four more cases unrelated to Fort Dix were reported in 1976 in Missouri, Wisconsin, and Minnesota [91]. The first cases of swine flu associated with exhibition pigs occurred in 1979-80. SIV similar to A/New Jersey/1976 was isolated from two people in Texas, both of whom had attended pig exhibitions [92].

A fatal case of pneumonia in a 5-year-old girl in Nevada in 1982 was described. Death was attributed to New Jersey/1976-like SIV infection [93].

In September 1988, the H1N1 swine flu virus was detected in a previously healthy 32-year-old pregnant woman in Wisconsin, who was hospitalized with pneumonia and died eight days later. Four days before being admitted to the hospital, the patient had visited a county hog fair where a flu-like illness was widespread among the animals [86,94,95,96].

New insight into SIV infections in humans emerged in 1991, when a caretaker of laboratory animals infected with SIVs in Maryland became ill and died of pneumonia. SIVs infections were confirmed serologically in four other laboratory workers [101].

In 1994, zoonotic transmission of SIV was also observed in Wisconsin between two laboratory research workers caring for pigs experimentally infected with A/Sw/IN/1726/88 [102,103]. Influenza viruses isolated from the workers were antigenically identical to the inoculum virus. Sequence analysis of the HA and NA genes of viruses isolated from each laboratory worker did not differ. In addition, sequencing of the remaining influenza gene segments did not indicate that viral reassortment had occurred. Although it was concluded that zoonotic transmission of SIVs does not require HA or NA gene mutations or viral reassortment, the potential remains and, therefore, human SIVs infections should be monitored for viral reassortment and associated mutations.

In 1995, in Minnesota, exposure to diseased pigs resulted in SIV infection and subsequent fatal pneumonia in a 37-year-old immunocompetent woman [104].

In 2006, SIV H3N2 was isolated from a hospitalized 7-month-old child in Canada. The strain was designated A/Canada/1158/2006 [108,109]. Nucleotide sequencing of all 8 RNA segments of the isolate showed that it was most closely related to A/swine/Ontario/33853/2005 (H3N2), which has the same triple reassortant (tr) genotype as the H3N2 subtype viruses that emerged in pigs in the United States in 1998 [109]. Serologic evidence of infection with the same strain was also found in 4 of 7 family members and 3 of 46 persons not related to the family.

In 2009, a 12-year-old boy was infected with SIV(tr)H3N2 in Kansas after visiting a county fair [110]. Pigs were suspected to be the carriers of the virus, as serologic testing of blood sera showed elevated titers against SIV A(H3N2), indicating prior infection [110,111].

IAVs of swine origin that infect humans are called variant IAVs, designated by the letter “v” after the subtype. Subsequently, beginning in 2011, there was a surge in cases of variant A(H3N2)v associated with exposure to pigs. For example, in 2011, two cases of febrile respiratory illness caused by variant SIV A(H3N2)v were described in the United States [120,121]. Sequencing showed that this virus contained seven genes derived from SIV (tr)A/H3N2, which has circulated in the US pig population since 1998, as well as the M gene from A(H1N1) pdm09. From 2011 to 2013, A(H3N2)v infection was detected in 340 people [123,124,125], one patient died. Most cases reported prolonged exposure to pigs prior to illness.

In 2016, outbreaks of A(H3N2)v were reported in Ohio and Michigan, and 18 zoonotic cases of IAV were detected [126]. All individuals had contact with pigs while visiting fairs. Also, viruses nearly identical to those detected in humans were isolated from pigs at these fairs, and the sequences of human virus gene segments were nested in monophyletic clades of swine viruses [127].

Zoonotic (swine-to-human) infections caused by IAV occurred in Brazil, in the state of Paraná, in 2020–2023. During the period, eight cases of human infection of IAV variants of swine origin were detected (6 mild and 2 severe cases, including 1 death). Three IAV subtypes that are zoonotic in swine (H1N1, H1N2, and H3N2) were detected in individuals with direct or indirect contact with these animals [141]. Five H1v IAVs were closely related to the pdm09 lineage, one H1v (H1N2v) grouped in clade 1B.2.3, and two H3v grouped in a clade consisting exclusively of Brazilian H3 swIAV (clade H3.1990.5.1). Internal gene segments were closely related to H1N1pdm09 isolated from swine.

Subsequently, from 2011 to 2023, more than 490 cases of variant IAV of both H1 and H3 subtypes were reported in the United States [122].

#### 3.2.2. Transmission of the Swine Influenza Virus to Humans in Europe

In 1958, the first serological evidence of SIV infection in humans was presented, associated with a 40-year-old woman from Czechoslovakia who had been in contact with pigs [78,79,80]. Epidemiologic analysis revealed probable human-to-human transmission, as five family members became ill with influenza-like symptoms and had serologic evidence of human SIV H1N1.

In March 1982, SIV with HA H1 was isolated from two sick adolescents in rural Bulgaria who had contact with each other and with animals in their backyard plots [78].

H1N1 SIVs were isolated from two people in Switzerland and one in the Netherlands in early 1986. The three viruses were closely antigenically related to each other and to the A/New Jersey/8/76 strain. The Swiss patients developed only mild symptoms, whereas the Dutch patient suffered from severe pneumonia. Two of the patients had close contact with diseased pigs [97].

In 1992–1993, three young children in the Netherlands were infected with the human–avian reassortant IAV H3N2. The children had no contact with pigs and suffered a mild respiratory illness [105,106].

Human cases of SIV infection were also observed in 2002 and 2008 in Switzerland and Spain, respectively. SIV H1N1 was isolated from two 50-year-old pig farmers [112,113,114].

There are reports of isolation of A(H1N1) SIVs from humans with influenza-like illness in Germany in 2007, 2010 and 2011 [115,116]. A comprehensive analysis of influenza A(H1N1) SIVs showed that the HA genes of these human isolates belonged simultaneously to two different clades, H1avN1 1C.2.2 and H1avN1 1C.2.1, belonging to the Eurasian lineage of avian influenza A(H1N1) in pigs. Antigen profiling revealed significant cross-reactivity between the two zoonotic H1N1 viruses and the human A(H1N1)pdm09 virus and some swine viruses, but no cross-reactivity to H1N2 and earlier human seasonal A(H1N1) viruses [116].

In 2019, a zoonotic infection with the Eurasian avian influenza A(H1N1) virus occurred in the Netherlands in a pig farmer. The virus was also detected in pigs raised on the farm [128].

In 2020, a zoonotic infection with influenza virus A/sw/H1avN1 1C.2.2 was again reported from Germany in a child who had close contact with pigs 3 days before the beginning of symptoms [142]. Sequence analysis showed that most HA antigenic sites were conserved between influenza A/sw/H1avN1 and A(H1N1)pdm09 viruses [143].

A severe case of zoonotic human infection with swine-origin IAV was reported in Denmark in November 2021 [144]. The sample was confirmed as positive for the A(H1N1)pdm09 strain and had greater similarity to SIVs than to other human strains. The patient handled live pigs, carcasses, and meat during the slaughter process, wearing personal protective equipment including gloves and a gown, but no mask. The patient was previously healthy, had no underlying medical conditions or immunodeficiency, and had received the recommended quadrivalent seasonal influenza vaccine in October 2021 [144].

#### 3.2.3. Transmission of Swine Influenza Virus to Humans in Asia

Three different SIV subtypes were responsible for four cases in Asia. In 1999, SIV H3N2 was isolated from a 10-month-old girl in Hong Kong [107]. Then again in Hong Kong in 2001, SIV H1N1 was isolated from a 4-month-old girl [117]. In the Philippines in 2004, SIV H1N2 was detected in a 25-year-old man, and then in 2005, SIV H1N1 was isolated from a 4-year-old boy in Thailand. All infected individuals had no direct contact with pigs. In both viruses, the HA genes were similar to the classic Asian and North American H1 SIVs, whereas the NA genes were more closely related to the European SIVs [118].

From 2015 to 2018, sporadic detections of humans infected with reassortant SIVs with gene pool of the A(H1N1)pdm09 were reported in China [129,130,131], and avian-like Eurasian SIV A(H1N1) with A(H1N1)pdm09 genes was reported to contribute to human infection [143].

In 2021, the influenza A(H1N2)v virus in Taiwan was first isolated from a 5-year-old girl who suffered from fever, runny nose, and cough. The isolated virus was denoted A/Taiwan/1/2021(H1N2)v. Whole genome sequencing and phylogenetic analysis revealed that A/Taiwan/1/2021(H1N2)v is a novel reassortant virus containing HA and NA gene segments derived from SIVs A(H1N2) that may have circulated in Taiwan for decades, and the other 6 internal genes (PB2, PB2, PA, NP, M, and NS) are derived from human A(H1N1)pdm09 viruses [145]

**Figure 5:**
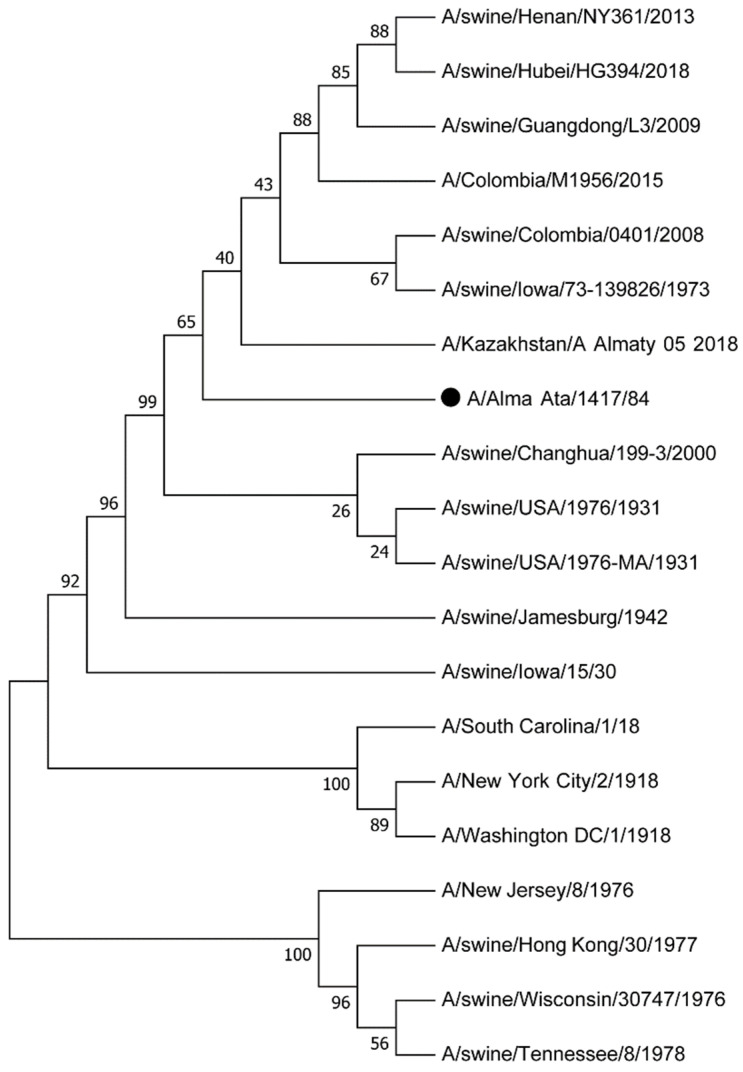
Molecular phylogenetic analysis of influenza virus A/Alma-Ata/1417/84 (indicated in the figure by a black dot) for hemagglutinin gen by Maximum Likelihood method. The phylogenetic tree was constructed using maximum likelihood with a bootstrap value of 1000. Bootstrap values are indicated at the node. The scale bar on the phylogenetic tree indicates the number of genetic changes.

Also, swine-like H1N1 viruses, together with H3N2 viruses, caused a seasonal increase influenza among the population in Almaty, Kazakhstan, in 1984 [98]. Three Almaty swine-like strains were isolated from lung and tracheal specimens from adults who died at the age of 46, 47, and 65 years from fulminant abdominal influenza with simultaneous damage to the cardiovascular and respiratory systems. All three had contact with pigs and lived in rural areas. A comparative analysis of the antigen structure of the Almaty and Bulgarian “swine-like” H1N1 strains, isolated from humans, with the reference viruses A/swine/Iowa/15/30, A/New Jersey/18/76 and A/duck/Alberta/35/76 revealed their practical identity in the HA gene with the virus A/swine/Iowa/15/30 and significant differences in the antigen structure of NA, which had greater similarity with the enzyme of the reference strain of the human H1N1 virus of 1978 circulation [99,100]. Information on the genetic sequence of the HA gene of the A/Alma-Ata/1417/84 strain is available in the international NCBI database under number S62154.1. Molecular phylogenetic analysis of the influenza virus A/Alma-Ata/1417/84 for the hemagglutinin gene with IAVs from the international NCBI database is presented in Figure 5. The closest IVs to this strain were strains circulating in pigs in 1930–2018—JX826516.1 (A/swine/Colombia/0401/2008), FJ752502.1 (A/swine/Changhua/199-3/2000), CY045740.1 (A/swine/USA/1976/1931), KY765314.1 (A/swine/Iowa/73-139826/1973), CY045748.1 (A/swine/USA/1976-MA/1931), AF091308. (A/swine/Iowa/15/30), CY026427.1 (A/swine/Jamesburg/1942), HQ877027.1 (A/swine/Guangdong/L3/2009), MW126699.1 (A/swine/Henan/NY361/2013), MW127183.1 (A/swine/Hubei/HG394/2018)—the homology percentage is 97.53–99.18%. In addition, in 1976–1978, In pigs, strains CY028187.1 (A/swine/Wisconsin/30747/1976), CY027523.1 (A/swine/Tennessee/8/1978) and CY084529.2 (A/swine/Hong Kong/30/1977) were detected—the percentage of homology is 88.9–89.13%. Periodically, in humans, from the moment of its appearance in 1918 to 2015, closely related IVs were detected—AF117241. (A/South Carolina/1/18), OR133599.2 (A/New York City/2/1918), OR382007.1 (A/Washington DC/1/1918), CY130118.1 (A/New Jersey/8/1976) and PP885588.1 (A/Colombia/M1956/2015), with homology from 89.32 to 92.7%. This may indicate the circulation of cH1N1 swine IVs to the present day.

The evolutionary history was inferred by using the Maximum Likelihood method based on the Hasegawa–Kishino–Yano model [146]. The tree with the highest log likelihood (−3643.67) is shown. The percentage of trees in which the associated taxa clustered together is shown next to the branches. Initial tree(s) for the heuristic search were obtained automatically by applying Neighbor-Join and BioNJ algorithms to a matrix of pairwise distances estimated using the Maximum Composite Likelihood (MCL) approach, and then selecting the topology with superior log likelihood value. The rate variation model allowed for some sites to be evolutionarily invariable ([+*I*], 55, 13% sites). The analysis involved 20 nucleotide sequences. All positions containing gaps and missing data were eliminated. There were a total of 1552 positions in the final dataset. Evolutionary analyses were conducted in MEGA7 [147].

In November 2009, SIVs A/H1N1 were isolated from sick people in Kazakhstan [119]. Subtyping in RT-PCR and analysis of nucleotide sequences of surface protein genes of isolates showed that the influenza infection was caused simultaneously by viruses A/H1N1pdm and the seasonal influenza agent A/H3N2. Genetic studies have shown that Kazakhstani isolates A(H1N1)pdm are 99.2–99.4% similar to the virus A/California/07/09 (H1N1)pdm in their HA and NA genes [119].

Triple reassortant A/H1N1pdm09 continues to circulate in various regions of Kazakhstan to this day, along with viruses A/H3N2 and two lineages of type B [25,26,27,132,133].

#### 3.2.4. Transmission of the Swine Influenza Virus to Humans in Australia

Australia was long considered free of enzootic SIVs due to strict quarantine measures and the use of biosecure breeding facilities. Since 2009, IAV H1N1pdm has emerged sporadically in Australian pig populations but has had no apparent adverse health effects on infected pigs and is therefore not considered a threat to human or animal health or a potential source of pandemic viruses [134].

However, in 2011, an outbreak of swine influenza in pigs occurred on three pig farms located in three different states in Australia, resulting in infection of animal care workers. Strains detected in humans were identical to the swine isolates. The transmission of SIV to humans in Australia highlighted the importance of monitoring SIVs in pigs and humans to reduce the risk of zoonotic viruses emerging [134].

In 2012, an outbreak of swine influenza in pigs in Australia resulted in SIV infection among farm workers [135]. Investigation of this outbreak led to the discovery of a number of genetically distinct divergent IAVs in pigs. Public health officials raised the possibility of zoonotic infections and the potential for a future pandemic.

In 2018, a 15-year-old girl was reported infected with SIV A(H3N2)v. Genetic analysis of the virus showed that all genes were similar to viruses that have circulated and been detected in pigs in Australia over the past decade. Investigation into the sources of exposure revealed that the girl had participated in an agricultural event (including contact with live animals) the day before illness onset and had contact with animals at school and at home. No other cases associated with this event have been identified [136,137].

#### 3.2.5. Transmission of the Influenza Virus A/H1N1pdm to Humans

In March 2009, an epizootic of swine flu occurred in the suburbs of Mexico City, during which the A/H1N1 virus was isolated. This virus was able to infect people and be transmitted from infected people to contact persons, first in Mexico City, and by April 2009 it had spread to the United States and Canada, and then to other countries on all continents. In connection with this, in June 2009, the WHO declared the beginning of the first influenza pandemic in the 21st century, caused by a new influenza pathogen of the H1N1pdm subtype, a triple reassortant between viruses of the European and American lines of pigs, avian influenza viruses, and a human virus [148,149]. From the “seasonal” human influenza virus, it “acquired” new sequences of the PB1 protein gene, from the avian IAV—the PB2 and PA genes, and from the SIV—the HA, NA, NP, M2, NS2 genes. The new strain of this virus was named A/California/07/09 (H1N1)pdm based on the place and time of isolation of this virus from a sick person. Such a complex combination of genomic fragments undoubtedly led to the emergence of a completely new phenotype, which was reflected in the clinical picture of diseases caused by this pathogen and in its biological properties, expressed, among other things, in the features of isolation of this virus [150].

In Europe, the first laboratory-confirmed case of the disease associated with the new influenza variant A(H1N1)pdm was registered in April 2009 in Spain. In May, the flu spread to all Western European countries. The highest number of fatalities in Europe was registered in France, Germany and the United Kingdom [151].

The vast majority of SIVs circulating in global pig populations were acquired from humans [152], and the 2009 IAV A(H1N1) pandemic resulted in rapid reverse zoonosis in pig populations. It has been shown that the 2009 H1N1pdm virus infected and caused disease in pigs without prior adaptation [153] and has been detected in pig herds worldwide [154]. Human-to-pig transmission (reverse zoonosis) of the pandemic H1N1pdm virus has even been detected in Australia and Norway, which were previously considered swine influenza-free [21,134]. Since the 2009 pandemic, H1N1pdm IAV has continued interspecies transmission between pigs and humans in both directions, further complicating the relationship between swine and human IAV [155].

In the Western Pacific region, cases of influenza A(H1N1)pdm were observed in New Zealand, Mexico, and Australia. In South American countries, cases were observed in Costa Rica, Paraguay, Cuba, Belize, and Guatemala. The highest mortality rate (up to 7.9%) was observed in Brazil [151,156].

In the South-East Asian region, the first cases of influenza A(H1N1)pdm were registered in July 2009 in South Korea, India, Thailand, Bangladesh [156] and Japan [157].

In some studies have found circulation of influenza A virus in pig populations in Kenya and Nigeria, including the presence of human influenza A(H1N1)pdm09 virus in pigs. In addition, human infections have been reported in these regions following contact with infected pigs [138,139,140]. In Sinegal, the phylogenetic analysis revealed that IAV were closely related to human IAV strains belonging to lines A/H1N1pdm09 and seasonal H3N2 [158].

In Russia, the epidemic of swine flu A/California/07/09 (H1N1)pdm began in September 2009 in the Far East in the city of Yuzhno-Sakhalinsk among schoolchildren aged 7–14 years, and then among the adult population [159]. Subsequently, the epidemic began to spread to other cities and regions. In total, the epidemic affected 49 cities in Russia and ended at the end of January 2010 [159]. During this time, 28 cases of death from laboratory-confirmed H1N1pdm influenza were registered.

Thus, IAV(H1N1)pdm has been reported worldwide with frequent reassortment with locally endemic viruses [160]. Two-sided transmission of IAV has accelerated the evolution of H1N1pdm, which has increased the severity of influenza virus infections since most people had no previous immunity against the swine virus.

Since its emergence in 2009, H1N1pdm09 has become established in the human population and continues to circulate as seasonal H1N1 IAV to date. Frequent transmission of H1N1pdm09 from humans to pigs, as well as the high prevalence of reassortants with co-circulating SIVs detected in pig herds in several countries, continue to pose a serious public health threat [18].

## 4. Discussion

IAVs are an integral part of a complex ecosystem that gives rise to viruses and zoonotic diseases and, being endemic in pigs, pose a public health risk. Although human infection with zoonotic influenza viruses is less common than infection with seasonal influenza viruses, there is global concern that these zoonotic viruses may acquire mutations in animals or humans and facilitate efficient animal-to-human transmission or sustained human-to-human transmission. Globally, pigs, as a major reservoir species, play a key role in the transmission of IAV between species and the emergence of zoonotic influenza and pandemic viruses. This was confirmed by the 2009 pandemic, which highlighted the role of pigs in the emergence of IAV with pandemic potential. In the swine reservoir, genetic mutation coupled with reassortment leads to the emergence of new strains of IAVs that cross species boundaries by shuffling gene segments between hosts (birds, pigs, humans) and may lead to the appearance of viruses with unpredictable transmissibility and pathogenesis [161].

Zoonotic transmission allows the introduction of new IAV strains into the human population, potentially causing the next influenza pandemic. The incidence of zoonotic infections increases when humans live in close contact with pigs or when exposed to them while attending agricultural fairs. For example, pig exhibits at agricultural fairs have become a source of SIV amplification; these unique pig–human interfaces have caused the majority of human cases of variant IAV infection in the United States [122,126,127,141].

People working on pig farms also play a special role in the mixing of IAVs, leading to reassortment and the emergence of new offspring strains with pandemic potential. By contacting animals, they are at risk of being among the first to be infected if the new virus becomes epizootic in pig herds, i.e., workers can serve as a link for the transmission of the virus to their communities [162].

Although SIVs can infect anyone, some people are at increased risk of complications from the infection. These include people younger than 5 years and older than 65 years, pregnant women, and people with weakened immune systems [163].

Based on the data we collected, the main epidemiological factors influencing the transmission of IAVs from pigs to humans contain all factors that increase interactions between humans and pigs. These include close contact with pigs or contaminated environments, exposure to high viral loads from infected pigs, exposure of farm workers in intensive pig production, exposure of workers and visitors to agricultural fairs, and industrialization of pig production. Environmental factors such as climate and land use change may also significantly influence the transmission of influenza viruses from pigs to humans, creating more opportunities for disease outbreaks and interspecies transmission. Thus, climate change significantly changes the interaction of pigs and people, affecting farm density and migration routes, thereby increasing the risk of disease transmission [70]. Changes in temperature, rainfall, and extreme weather events associated with climate change may disrupt livestock production systems, causing changes in farming practices and potentially leading to farms becoming larger or being moved geographically. These changes, combined with climate-induced changes in wildlife migration patterns, may increase the potential for zoonotic diseases to spread.

To minimize the risk of reverse zoonosis of the influenza virus, it is necessary first of all to vaccinate both people (pig workers) and susceptible animals, strictly observe hygiene rules and limit contact with animals in case of illness. In particular, it is necessary to wash hands frequently, avoid touching the face and keep a distance from animals if symptoms of influenza appear. In addition, measures to prevent the introduction and spread of diseases among animals are necessary, especially at livestock shows and fairs. This includes pre-show preparation, careful management during the fair, and isolation and monitoring after the show. Key aspects include minimizing contact with animals, cleaning and disinfection of equipment, and observing hygiene rules [164].

Therefore, surveillance of both pigs and humans, particularly those at increased risk of complications from infection due to exposure at the animal–human interface, is critical in the fight against emerging zoonotic SIVs. Limiting human–pig contact can help prevent severe infections and reduce the public health burden associated with the SIV variant and the risk of cross-species transmission events.

The “One Health” concept, which has gained worldwide recognition, is based on the idea that human health is linked to animal and environmental health [165]. The main focus of the concept is to enhance cooperation and communication between the agricultural and health sectors. The World Health Organization (WHO) and the World Organisation for Animal Health (WOAH) also work closely together. Together, WHO and OIE regularly analyze available data to identify and characterize viruses that pose a potential pandemic threat, as well as to select candidate viruses for vaccine development and pandemic preparedness [166].

Improved surveillance of swine and human populations for emerging influenza viruses, along with wider seasonal vaccination coverage among agricultural workers, health care workers and the general public, are critical strategies in the fight against emerging zoonotic SIVs and reduce the risk of reassortment events [167,168,169].

## 5. Conclusions

Diverse IAVs circulating and continuing to evolve in animal hosts may provide a source of new IAVs to which humans are generally not susceptible and pose a public health and pandemic risk if they adapt to human transmission [170].

The greatest risk posed by zoonotic infections is the opportunity they provide for reassortment between human and swine strains of IAV. The 2009 pandemic of swine-origin IAV highlighted the importance of understanding the pathogenesis, transmission, and evolution of SIVs in both humans and pigs.

Although swine workers are primarily exposed to SIVs, influenza transmission is limited due to the restricted human workforce and biosecurity measures in commercial establishments. The increased risk of SIV transmission from infected pigs to humans occurs at agricultural shows where many people come into contact with live pigs. Keeping naive animals with infected pigs creates conditions for mixing different IAVs and the possibility of viral outbreaks [93].

Considering the potential of the pig as a “mixing vessel” where both avian and human influenza viruses can undergo reassortment, the emergence of new viral diversity may occur. The epidemiology of swine influenza is of utmost importance worldwide to better understand the potential health risks to pigs and humans from mutated or reassortant viruses [159].

Although certain measures exist to minimize this risk, such as vaccination of pigs against IAVs [171] and reduction in the duration of pig exhibitions at agricultural fairs [94], ongoing active surveillance for influenza in pig and human populations is required to predict and prevent interspecies transmission events associated with pandemic risk.

## Figures and Tables

**Table 1:** Continents and countries with cases influenza viruses’ transmission from pigs to humans.

Year	Continents and Countries	Influenza A Virus Transmitted from Swine to Humans	References
1958	Europe: Czechoslovakia	H1N1	[78,79,80]
1970–1980	America: Tennessee, Wisconsin, Virginia, New Jersey, Missouri, Minnesota, Texas	H1N1	[78,81,82,83,84,85,86,87,88,89,90,91,92]
1981–1990	America: Wisconsin, Nevada Europe: Bulgaria, Switzerland, Netherlands Asia: Kazakhstan	H1N1, cH1N1	[78,86,93,94,95,96,97,98,99,100]
1991–2000	America: Maryland, Wisconsin, Minnesota Europe: Netherlands Asia: Hong Kong	H1N1, H3N2	[101,102,103,104,105,106,107]
2001–2010	America: Canada, Kansas Europe: Switzerland, Spain, Germany Asia: Hong Kong, Philippine, Thailand, Kazakhstan	H1N1, (H1N1)v, (H1N1)pdm09, H1N2, H3N2, (H3N2)v	[108,109,110,111,112,113,114,115,116,117,118,119]
2011–2020	America: Indiana, Pennsylvania, Atlanta, Hawaii, Illinois, Maryland, Michigan, Minnesota, Ohio, West Virginia, Wisconsin, New Jersey and Iowa Europe: Germany, Netherlands Asia: China, Kazakhstan Australia: Queensland, Melbourne, Victoria, Western Australia, Perth, South Australia Africa: Kenya, Nigeria	H1N1, (H1N1)pdm09 (H3N2)v, H1N2, H3N2, (H3N2)v	[25,26,27,116,120,121,122,123,124,125,126,127,128,129,130,131,132,133,134,135,136,137,138,139,140]
2020–2024	America: Brazil, Paraná Europe: Germany, Denmark Asia: Taiwan	H1N1, (H1N1)v, (H1N1)pdm09, A/sw/H1avN1 1C.2.2, (H1N2)v (H3N2)v	[25,26,27,132,133,141,142,143,144,145]

## Data Availability

No new data were created or analyzed in this study. Data sharing is not applicable to this article.
